# Pre-analytic factors and initial biomarker levels in community- acquired pneumonia patients

**DOI:** 10.1186/cc13411

**Published:** 2014-03-17

**Authors:** A Kutz, E Grolimund, B Mueller, P Schuetz

**Affiliations:** 1 Kantonsspital Aarau, Switzerland

## Introduction

Blood biomarkers are increasingly used to diagnose, guide therapy in, and risk-stratify community-acquired pneumonia (CAP) patients in emergency departments (EDs). How pre-analytic factors affect these markers' initial levels in this population is unknown.

## Methods

In this secondary analysis of consecutive ED patients with CAP from a large multicentre antibiotic stewardship trial [1], we used adjusted multivariate regression models to determine the magnitude and statistical significance of differences in mean baseline concentrations of five biomarkers (procalcitonin (PCT), C-reactive protein (CRP), white blood cell count, proadrenomedullin (ProADM), copeptin) associated with six pre-analytic factors (antibiotic or corticosteroid pretreatment, age, gender, chronic renal failure or liver insufficiency).

## Results

Of 925 CAP patients (median age 73 years, 58.8% male), 25.5% had antibiotic pretreatment, 2.4%, corticosteroid pretreatment, 22.3% chronic renal failure, and 2.4% chronic liver insufficiency. Differences associated with pre-analytic factors averaged 6.1 ± 4.6%; the three largest statistically significant changes (95% Cl) were: PCT, +14.2% (+2.1 to +26.4%, *P *= 0.02) in patients with liver insufficiency; ProADM, + 13.2% (+10.2 to +16.1%, *P *< 0.01) in patients with age above median; and CRP, -12.8% (-25.4 to -0.2%, *P *= 0.05) with steroid pretreatment.

## Conclusion

The influence of pre-analytic factors on the examined biomarkers is marginal and not clinically relevant. Our observations reinforce the concept ofusing biomarkers in algorithms with widely- separated cutoff values and overruling criteria considering the entire clinical picture, without adjustment for pre-analytic factors.
